# Experimental Study on the Mechanical Properties and Compression Size Effect of Recycled Aggregate Concrete

**DOI:** 10.3390/ma14092323

**Published:** 2021-04-29

**Authors:** Yubing Du, Zhiqing Zhao, Qiang Xiao, Feiting Shi, Jianming Yang, Peiwei Gao

**Affiliations:** 1 Department of Civil and Airport Engineering, College of Civilaviation (College of Flight), Nanjing University of Aeronautics and Astronautics, Nanjing 210016, China; 2 College of Civil Engineering, Yancheng Institute of Technology, Yancheng 224051, China; shifeiting150632@163.com (F.S.); yjm_kk@163.com (J.Y.); 3 Jiangsu Collaborative Innovation Center for Ecological Building Materials and Environmental Protection, Yancheng 224051, China; 4 Global Institute of Software Technology, School of Art and Architecture, Suzhou 215163, China; zzq18801581991@126.com; 5 Jiangsu Water Conservancy Construction Engineering Co. Ltd., Yangzhou 225004, China; jssjxq1981@163.com

**Keywords:** aggregate substitution ratio, recycled aggregate concrete, basic mechanical properties, size effect, experimental study

## Abstract

To explore the basic mechanical properties and size effects of recycled aggregate concrete (RAC) with different substitution ratios of coarse recycled concrete aggregates (CRCAs) to replace natural coarse aggregates (NCA), the failure modes and mechanical parameters of RAC under different loading conditions including compression, splitting tensile resistance and direct shear were compared and analyzed. The conclusions drawn are as follows: the failure mechanisms of concrete with different substitution ratios of CRCAs are similar; with the increase in substitution ratio, the peak compressive stress and peak tensile stress of RAC decrease gradually, the splitting limit displacement decreases, and the splitting tensile modulus slightly increases; with the increase in the concrete cube’s side length, the peak compressive stress of RAC declines gradually, but the integrity after compression is gradually improved; and the increase in the substitution ratio of the recycled aggregate reduces the impact of the size effect on the peak compressive stress of RAC. Furthermore, an influence equation of the coupling effect of the substitution ratio and size effect on the peak compressive stress of RAC was quantitatively established. The research results are of great significance for the engineering application of RAC and the strength selection of RAC structure design.

## 1. Introduction

The continuous development of urbanization and the alternation of old and new buildings have given rise to the problem of considerable construction and demolition (C&D) waste. How to dispose and reuse the C&D waste in an environmentally friendly way has attracted significant attention. In response to this issue, experts in related fields have proposed a kind of recycled aggregate concrete (RAC) which is formed by partly or completely replacing the natural coarse aggregates (NCAs) in ordinary concrete with the recycled concrete aggregates (RCAs) obtained by crushing and screening the concrete C&D waste. During the last 20 years, the recycling of C&D waste has emerged as a socioeconomic priority, mainly in developed countries, and in the present decade, developing countries are also gradually adopting this priority. Technologies for the separation and recovery of C&D waste are well established, readily accessible and generally inexpensive [1]. Additionally, reprocessing C&D waste brings considerable environmental benefits, such as reducing the consumption of virgin aggregate, reducing quarries, and it is more conducive to the environment and ecological balance. Nayana et al. evaluated that recycled aggregates reduce greenhouse gas emission by 23–28% compared to natural aggregates [2,3]. As the quality of recycled aggregate is continuously improving and maintaining stable and relevant technical standards, the proportion of recycled aggregates replacing natural aggregates in fresh-mixed concrete can be further increased [4,5], recycled aggregates may be applied in high-performance concrete [6], and their properties were suitably modelled by normal distributions [7].

With regard to research on the basic mechanical properties of RAC, Katz [8] and Achtemichuk [9] studied the influence of the crushing time of recycled aggregate on the strength of RAC. The results showed that the strength of RAC was not affected by the failure time of recycled aggregate. A.K. Padmini [10] also studied the impact of recycled aggregate on the strength of RAC. Many scholars [11,12,13,14,15] have studied the mechanical properties of RAC, which differ in terms of the specification of recycled material, substitution ratio, and porosity. Xiao [16,17] systematically studied the tensile and compressive stress and structural application of RAC. Moreover, Chen [18] adopted a pseudo-triaxial testing machine to study the multi-axis mechanical properties of RAC and put forward the corresponding failure criterion and constitutive relation.

The size effect of concrete defines the mechanical property of concrete as a variable that varies according to the geometric size of concrete, and studies on the size effect of concrete are conducive to further understanding the impact of small-size structure tests on actual structure research [19,20]. By analyzing the compressive strength of the concrete cubes of three different sizes, Neville [21] found that the smaller the size, the more obvious the size effect of concrete’s compressive strength. Jin [22,23] carried out numerical analysis and theoretical research on the impact of the size effect and strain rate coupling on the mechanical properties of ordinary concrete. Furthermore, Su [24] studied the size effect of the compressive strength of ordinary concrete and high-strength concrete and proposed the size effect law equation of concrete with different strength grades; the results showed that high-strength concrete displays an obvious compressive strength size effect. The literature [25,26] includes qualitative research on the compression size effect of RAC with different substitution ratios. Nevertheless, the above research merely analyzed the basic mechanical properties in terms of compression and tension loading modes, whereas the shear mechanical property of RAC has rarely been reported. Furthermore, the size effect of RAC has only been qualitatively studied. Therefore, the study of shear mechanical properties and quantitative analysis of the compression size effect of RAC are important novelties, which are of great significance for the engineering application of RAC and the strength selection of RAC structure design.

In this paper, the basic mechanical properties and compression size effects of RAC with different aggregate substitution ratios were experimentally studied. Considering the impacts of factors such as five substitution ratios of recycled aggregate (0%, 25%, 50%, 75%, and 100%), three loading modes (compression loading, tension loading, and shear loading) and three side lengths (70 mm, 100 mm, 150 mm), with the failure modes and mechanical parameters of RAC obtained by hydraulic servo-motor and direct shear apparatus under different loading modes, the basic mechanical properties and compression size effect of RAC were systematically analyzed from qualitative and quantitative perspectives, respectively.

## 2. Materials and Methods

### 2.1. Materials and Mix Proportion Design of Specimens

In this paper, an experimental study on the basic mechanical properties and compression size effect of RAC with different substitution ratios is presented. The aggregate substitution ratios of RAC employed in this study were 0%, 25%, 50%, 75%, and 100%, among which the concrete with a substitution ratio of 0% refers to ordinary concrete. The raw materials of these RAC specimens included cement, fine aggregate, coarse aggregate, and tap water; the additives in the raw material were not taken into account. The cement was ordinary Portland cement P·O 42.5 from China United Cement Corporation in Jiangsu province; the fine aggregates were natural river sand produced locally whose fineness modulus is 2.5 and apparent density is 2650 kg/m^3^.

**Figure 1:**
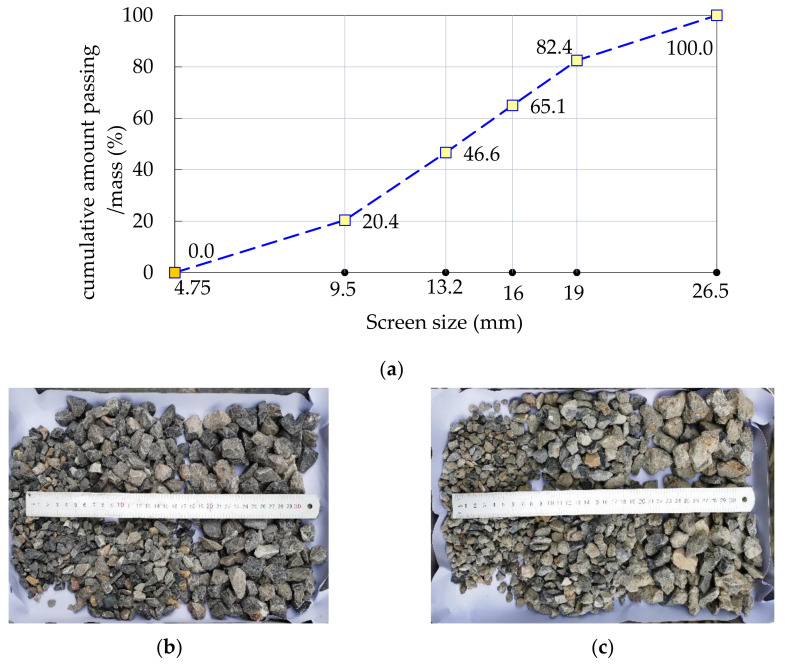
Screening and composing gradation of coarse aggregates for: (**a**) the grading curve of natural coarse aggregate used; (**b**) the appearance of natural coarse aggregates with continuous gradation; and (**c**) the appearance of coarse recycled concrete aggregates with continuous gradation.

**Figure 2:**
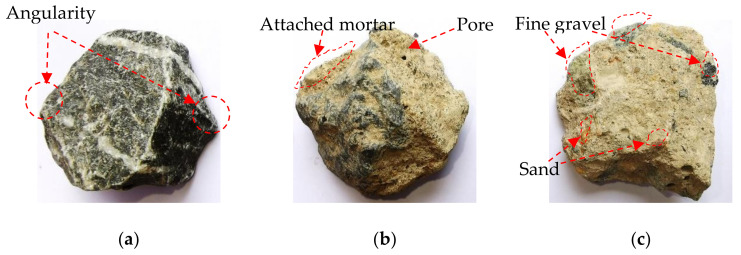
Appearance morphology of natural coarse aggregate and coarse recycled concrete aggregate: (**a**) natural coarse aggregate; (**b**) coarse recycled concrete aggregate; and (**c**) mortar block.

Many previous studies have presented that the compressive strength of recycled concrete mixed with a certain proportion of fine recycled concrete aggregates (FRCAs) is greatly reduced due to the large water absorption rate of FRCAs [27,28,29]. Additionally, the adverse effect of FRCAs on concrete is greater than that of coarse recycled concrete aggregates (CRCAs) [30]. In this study, only CRCAs were used to replace natural coarse aggregates (NCAs) in different proportions. The NCA used was limestone gravel, purchased from the local sand and gravel market, and its grading curve is shown in Figure 1a. Recycled aggregates were provided by Jiangsu Dongying Municipal Engineering Co., Ltd., Yancheng, China, which were obtained from a broken cement concrete pavement slab as a master material and crushed by a jaw crusher. In this study, the recycled aggregates were further cleaned, dried, and graded by square hole sieves with five different pore sizes, and then weighed and mixed with reference to the gradation of NCA. Therefore, CRCAs and NCAs have similar particle gradation characteristics, as shown in Figure 1b,d. Figure 2 shows the typical morphological characteristics of NCAs and CRCAs. The NCAs have obvious angular properties, and most of the CRCAs are bonded with a few old mortar blocks, while a few of the CRCAs are the blocks formed by the adhesion of some fine aggregates the old cement slurry. The particle sizes of the two types of coarse aggregates ranged between 4 mm and 16 mm, and their physical characteristics are listed in Table 1. Due to the porous mortar attached to the surface of the old aggregate and the mortar block with relatively weak strength, the water absorption and crushing index of the CRCAs are obviously higher than those of the NCAs.

When the substitution ratio of CRCA is 0%, this means that the concrete is ordinary concrete. In the present study, the design strength of ordinary concrete was 30 MPa. The design and calculation of the mix proportion were conducted according to the Specification for Mix Proportion Design of Ordinary Concrete (Chinese Standard JGJ 55-2011) [31]. Before pouring recycled concrete, the CRCAs were in the saturated surface-dry (SSD) moisture state which was processed according to the method of reference [32]. By applying the method of the equal mass substitution ratio and by replacing the NCA with CRCA, the mix proportions of concrete when the substitution ratios of the CRCA were 0%, 25%, 50%, 75%, and 100%, as shown in Table 2.

According to the calculation results of the mix proportion of RAC with different aggregate substitution ratios presented in Table 2, the cement, fine aggregate, natural aggregate, and recycled aggregate that had been weighted were successively poured into the mixer and mixed evenly. The water was subsequently poured into the mixer for stirring, and after this was completed, the mixed material was poured into the test mold. Then, the test mold was placed on the vibration table for vibrating. One day later, the mold was removed, and the specimens were immediately put into a standard curing room with humidity over 95% and temperature 20 ± 2 °C for 28 days, after which the test was carried out.

### 2.2. Experimental Scheme and Loading Devices

In this paper, the basic mechanical properties and size effect of five kinds of RAC with different substitution ratios were studied. The loading modes employed for analyzing the basic mechanical properties included uniaxial compression, uniaxial tension, and shear, among which uniaxial tension was completed by splitting tensile loading. For investigating the size effect of concrete, considering the stress state of concrete in practical engineering, this research only conducted an experimental study on the compression size effect of RAC.

According to the Standard for Test Method of Mechanical Properties on Ordinary Concrete (Chinese Standard GB/T 50081-2019) [33], the dimensions of the RAC specimens under compression and tension loading were 100 mm × 100 mm × 100 mm. As for the shear loading, considering the size limitation of the shear box of the actual loading equipment and concrete shear loading size in the relevant literature [34], 205 mm × 205 mm × 150 mm rectangular specimens were adopted. The shear section size was 205 mm × 205 mm. For the compression size effect of RAC, three concrete cubes with side lengths of 70 mm/100 mm/150 mm were designed by referring to an experimental study on the size effect of concrete from the relevant literature [24]. In view of the randomness and discreteness of concrete materials, three specimens were designed for each loading mode, and the mean value of the three specimens was used for experimental analysis.

In this paper, the compression loading of RAC was completed by the hydraulic servo-motor; the tension loading was realized by the combination of a hydraulic servo-motor and splitting tensile mold; and the shear loading was completed with the direct shear apparatus. The independent load transducers of the hydraulic servo-motor and the direct shear apparatus were able to meet the requirements of load measurement. The deformation was measured by the strain acquisition instrument and the independent deformation acquisition system. The load and deformation measurements were also able to meet the test requirements.

The loading of RAC with different aggregate substitution ratios was realized by a load–displacement hybrid control. First, by means of the load control, with a 20% maximum strength as the preloading value, the loading and unloading processes were repeated three times to control the influence of the gap between the loading end of the equipment and the loading surface of the specimen on the test results. Then, the loading was started by displacement control at a speed of 1 mm/min. The load and deformation data were collected at the same time, and the loading was terminated when the specimen was damaged. In order to control the friction between the loading end of the equipment and the loading surface of the specimen, this study used the combination of three layers of polyethylene plastic film and mineral butter mentioned in Reference [8], so that the test requirements could be satisfied.

## 3. Analysis of Experimental Results

### 3.1. Basic Mechanical Properties

#### 3.1.1. Failure Mode

**Figure 3:**
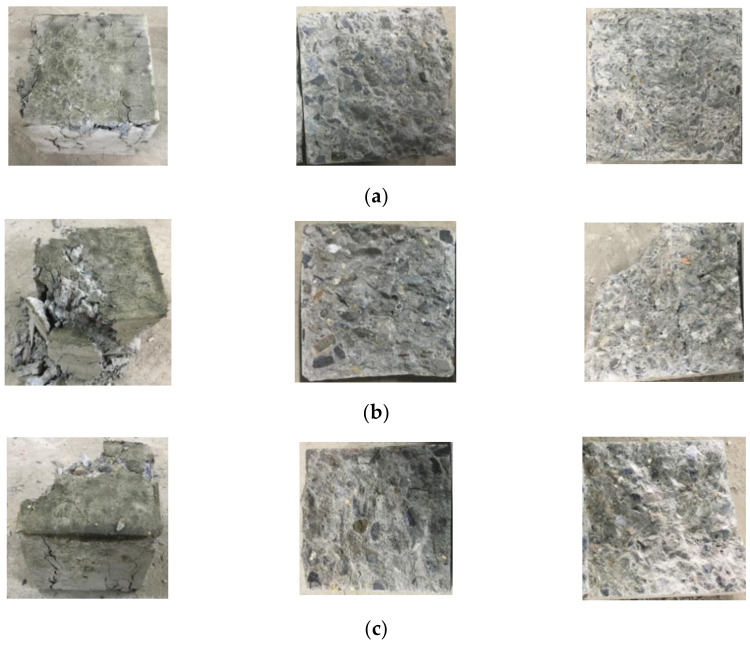
Basic failure modes of recycled aggregate concrete with different substitution ratios (from left to right are the results of compression, tensile and shear loading tests): (**a**) 0% substitution ratio of recycled aggregate; (**b**) 50% substitution ratio of recycled aggregate; and (**c**) 100% substitution ratio of recycled aggregate.

According to the experimental scheme of the basic mechanical properties of RAC with different aggregate substitution ratios, the failure modes of the concrete under compression, tension, and shear loading were obtained. Based on the study of the failure modes of RAC, the influence of the substitution ratio of recycled aggregate on the basic mechanical properties of RAC could be analyzed from a macroscopic perspective. In view of the length of the paper, the failure modes of RAC were analyzed at the aggregate substitution ratios of 0%, 50%, and 100%, as shown in Figure 3.

As can be observed from Figure 3, the compression failure mode of RAC is slightly impacted by the substitution ratio of recycled aggregate. Under the influence of Poisson’s ratio effect, the tensile strain is formed on the unloaded surface of the specimen under compression. When the tensile strain reaches the limit of tensile strain of concrete, the specimen is destroyed. The difference lies in the fact that, with an increase in the substitution ratio of recycled aggregate, the fine cracks formed on the non-loading surface of RAC gradually decrease, and a large amount of recycled aggregate falls off. For the tensile test of RAC under splitting tensile loading, when the substitution ratio of recycled aggregate is 0%, the splitting tensile section of concrete is relatively flat; when the substitution ratio is not 0%, a small number of coarse aggregates fall from the splitting tensile section of concrete, in which case the splitting tensile section is rather uneven. As for the failure mode of RAC under shear loading, when the substitution ratio of recycled aggregate is 0%, the shear loading surface of concrete is flat and the amount of concrete debris is small compared with the concrete containing recycled aggregates; the shear failure section of concrete with recycled aggregates is uneven, and there are many concrete fragments. Regardless of the loading mode, the concrete with recycled aggregates not only suffers from the failure of the cementing layer, but also the failure of a certain amount of recycled aggregate. In contrast, the ordinary concrete only displays the failure of the cementing layer.

**Figure 4:**
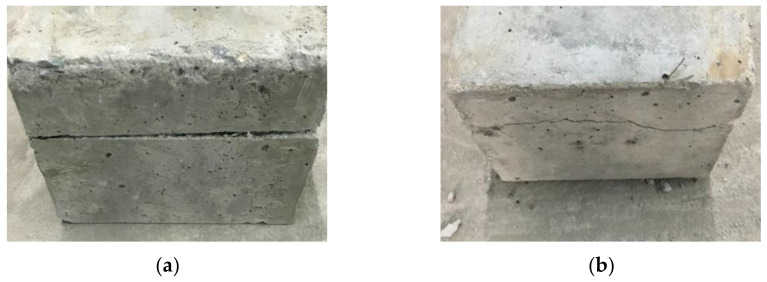
Shear failure mode of the recycled aggregate concrete for: (**a**) surface of the concrete specimen along the vertical shear direction; and (**b**) surface of the concrete specimen along the horizontal shear direction.

In order to further study the shear failure mode of RAC, a substitution ratio of recycled aggregate of 50% was used for the analysis, as shown in Figure 4 [34].

It can be observed in Figure 4 that a straight crack formed on the surface of the RAC specimen along the vertical shear direction. This crack was caused by the interaction between the upper and lower shear boxes of the direct shear instrument. Along the horizontal shear direction, a fluctuating but roughly straight crack formed on the specimen surface. The main reason for this is the randomness and discreteness of concrete failure. Specifically, since the specimen surface along the horizontal shear direction is not constrained by the load, the shear crack is rather fluctuating. Nevertheless, due to the constrains from the upper and lower shear boxes acting on the vertical shear surface of the specimen, the horizontal crack stays roughly straight.

#### 3.1.2. Stress–Strain Curve

**Figure 5:**
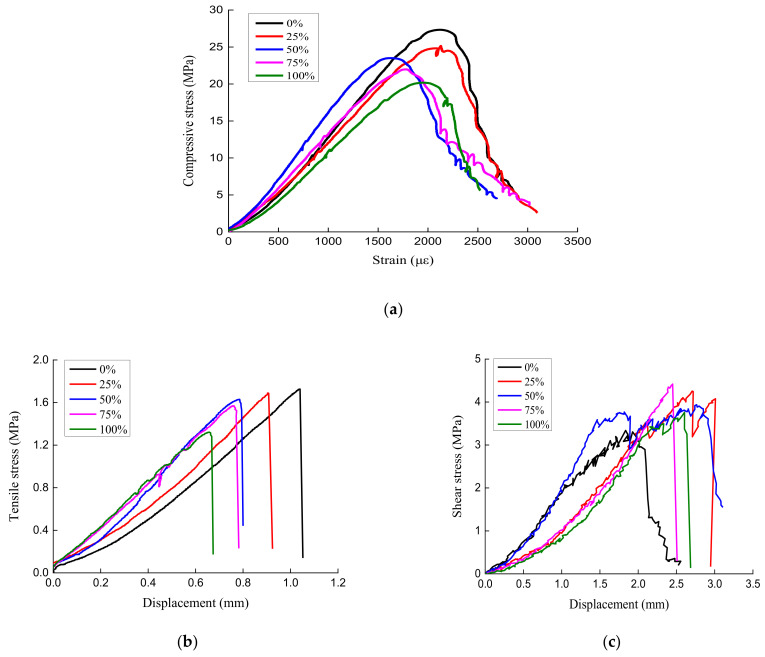
Stress–strain curves of recycled aggregate concrete for: (**a**) compression loading; (**b**) tension loading; and (**c**) shear loading.

The stress–strain curves of RAC under compression, tension, and shear loading were obtained based on the experimental scheme of the basic mechanical properties of RAC with different substitution ratios, as shown in Figure 5.

It can be seen from Figure 5 that the development of the compressive stress–strain curves of specimens with different substitution ratios of recycled aggregate have similar development patterns and show obvious continuity and smoothness. The replacement of NCA by CRCA has a certain influence on the characteristic value of the stress–strain curve of concrete. As shown in Figure 5a, the compressive stress–stain curve can be divided into three stages, namely, the elastic stage, elastic-plastic stage, and descending stage. Moreover, the elastic modulus of RAC is significantly lower than that of ordinary concrete when the latter part of the elastic stage (straight line) of the stress–strain curve is taken as the value point of the elastic modulus. The peak compressive stress and the descending section of RAC is always below that of ordinary concrete. This indicates that increasing the proportion of CRCA replacing NCA reduces the strength and brittleness of concrete. By analyzing the tensile stress–displacement curve of RAC under splitting tensile loading, it could be found that the substitution ratio of recycled aggregate has no effect on curve shape. However, with the increase in the recycled aggregate substitution ratio, the splitting limit displacement decreases, and the splitting tensile modulus slightly increases. The large-scale replacement of natural aggregate by CRCA increases the loss of the splitting tensile strength of concrete. Furthermore, the concrete breaks right after the splitting tensile stress of RAC reaches its peak, exhibiting obvious brittle failure features. Generally, the peak splitting tensile stress of RAC declines progressively as the substitution ratio of recycled aggregate rises. Through analyzing the shear stress–displacement curve of RAC with different aggregate substitution ratios, it could be found that the substitution ratio of recycled aggregate has no effect on the curve shape. The shear stress of ordinary concrete is lower than that of RAC. While the substitution ratio of recycled aggregate is 75%, 25%, 50% and 100%, the shear stress of RAC decreases gradually. When the substitution ratio of recycled aggregate is 75%, the shear stress of RAC is the largest. The effect of the substitution ratio of recycled aggregate on shear stress is different from that of compression and tensile stress.

#### 3.1.3. Characteristic Value of Stress

With the peak stresses that were obtained from the stress–deformation curves of RAC with different substitution ratios under compression, tension, and shear loading modes, the impact of substitution ratios of recycled aggregate on the peak stress of RAC, as well as the relationship between the substitution ratio and the variation of peak stress, was studied. The results are given in Table 3.

**Figure 6:**
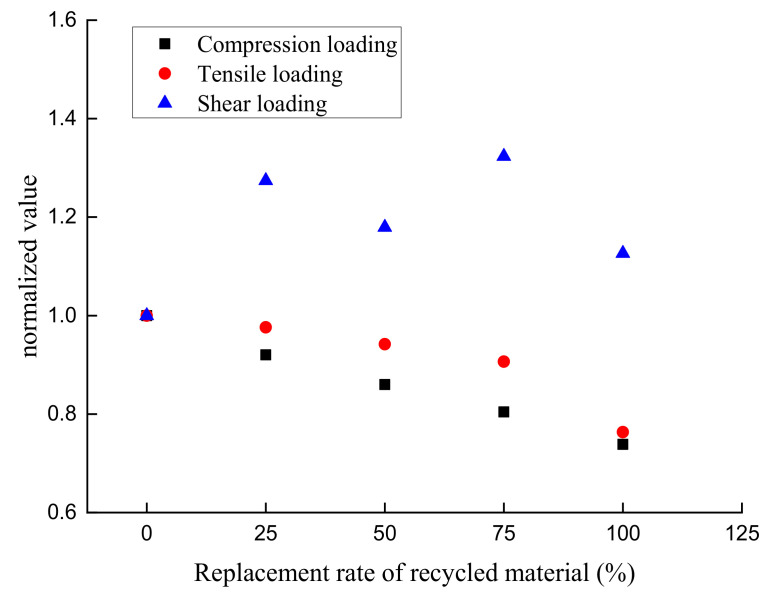
Correlation between the substitution ratio and normalized values.

To better analyze the influence of the substitution ratio of recycled aggregate on the peak compressive, tensile, and shear stresses of RAC, the correlation between the substitution ratio and peak stress of RAC was obtained from Table 3. If the normalized value is defined as the ratio of the peak stress of RAC to the peak stress of ordinary concrete, the normalized values of RAC with different ratios of CRCA replacing NCA is calculated according to the data in Table 3, as shown in Figure 6.

For different loading conditions, the strength of recycled concrete is expressed in the form of error bar. According to the analysis, the error range of the recycled concrete strength test value is controlled within 10%, which meets the research requirements. The analysis of Table 3 and Figure 6 shows that, as the substitution ratio of recycled aggregate rises, the peak compressive stress and the peak tensile stress of RAC both decline gradually, displaying a linear relationship between the substitution ratio of recycled aggregate and the influence coefficients of peak compressive stress and peak tensile stress. This is mainly related to the physical properties of the recycled aggregate. Specifically, compared with natural aggregate, there are more minor injuries on the surface of recycled aggregate [35], and the cube compressive strength for recycled aggregate is lower, so the peak compression and tensile stresses of the concrete decrease with an increasing substitution ratio of recycled aggregate. In this study, the maximum reduction in peak compressive stress and peak tensile stress of RAC is 26.16% and 23.70%, respectively, from the impact of the substitution ratio of recycled aggregate. Nevertheless, the change rule of peak stress of RAC by the influence of the substitution ratio under shear loading is different from those under compression or tension loading. It is primarily because the shear loading mode has a different failure mechanism to compression and tension loading modes. Under the action of shear loading, the specimen suffers from transverse shear failure. The peak shear stress of specimens is mainly provided by the chemical adhesive force, Van der Waals force, and mechanical interaction at the shear interface. Before shear cracking, cohesion is the dominant factor of shear strength. After shear cracking, aggregate interlock is one of the principal mechanisms of shear transfer across cracks. In this case, volume and characteristics of coarse aggregates and the strength of the mortar in concrete are likely to have a significant influence on the aggregate interlock [36]. The research results on the shear behavior of RAC are not uniform. Some studies indicated that the shear strength of RAC is slightly weaker than ordinary concrete (NC). Some studies also found that the shear strength of RAC was not significantly different from NC. However, a few studies believed that the shear strength of RAC is slightly higher than NC [37]. The experimental results of this study showed that the shear strength of RAC was higher than that of NC, but the increase in shear stress showed an unstable change with the increase in the aggregate substitution ratio. The reason is that the RCA surface mortar layer should have a high strength, but the surface mortar layer has micro pores, micro cracks and the uncertainty of adhesion strength with aggregate, which makes the shear test results have a certain degree of dispersion.

Researchers’ opinions [38,39] are divided on the influence law of peak compressive stress of RAC in terms of the substitution ratio of recycled aggregate. Some believe that the peak compressive stress of RAC gradually decreases with an increasing substitution ratio of recycled aggregate, whilst others think that it increases. The reason for this disagreement may lie in the different sources of recycled aggregate. If the strength of the source concrete of recycled aggregate is stronger than that of the RAC, then the peak compressive stress of the RAC will be higher than that of ordinary concrete; otherwise, the result will be the opposite. At the same time, parameters like the physical characteristics and particle sizes of recycled aggregate have a great impact on the peak compressive stress of RAC [35,40]. For example, the water-absorbing capacity of recycled aggregate can easily affect the water–binder ratio of RAC. When concrete is mixed with recycled aggregate without water absorption treatment, the water–binder ratio is reduced due to the increased water absorption of recycled aggregate than that of natural aggregate, and the strength of concrete will be improved to a certain extent. This paper agrees with the opinion that the peak compressive stress of RAC decreases progressively with a rising substitution ratio of recycled aggregate. According to references [16,17], the variation rule of the peak tensile stress of RAC impacted by the substitution ratio of recycled aggregate is as follows: with an increase in the substitution ratio of recycled aggregate, the peak tensile stress of RAC gradually decreases, which is similar to the conclusion of this paper. The peak shear stress of RAC is higher than that of ordinary concrete under the action of shear loading, which is mainly because the failure mechanism of concrete under shear force is different from that of concrete under compression and tension.

The literature [41] has proposed the relationship between the peak compressive stress, peak tensile stress, and peak shear stress of ordinary concrete. Equation (1) shows the correlation between the peak compressive stress and peak tensile stress of ordinary concrete, and Equation (2) shows the correlation between the peak compressive stress and peak shear stress of ordinary concrete:

(1) $$f_{t}=0.19f_{\mathrm{cu}}^{0.75}$$

(2) $$\tau =0.38f_{\mathrm{cu}}^{0.57}$$

where *f*_t_ is the peak tensile stress of concrete, *f*_cu_ is the peak compressive stress of concrete, and τ is the peak shear stress of concrete.

**Figure 7:**
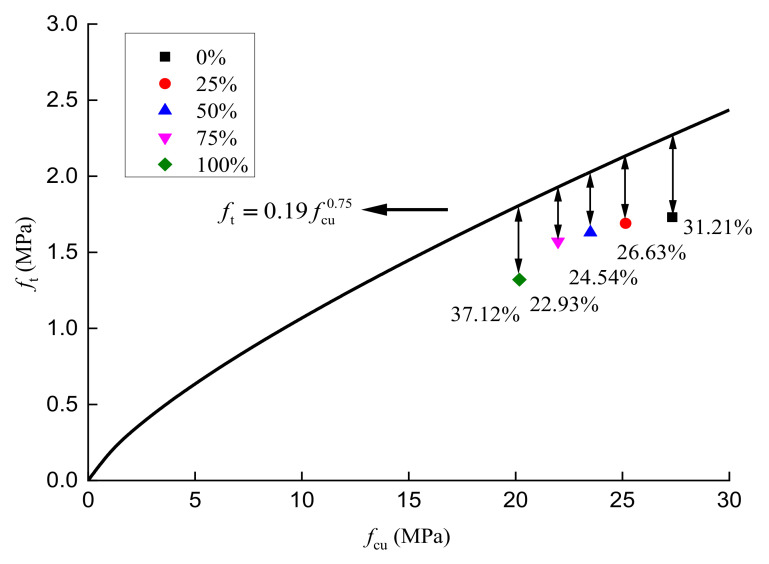
Correlation between the peak compressive stress and peak tensile stress of concrete.

**Figure 8:**
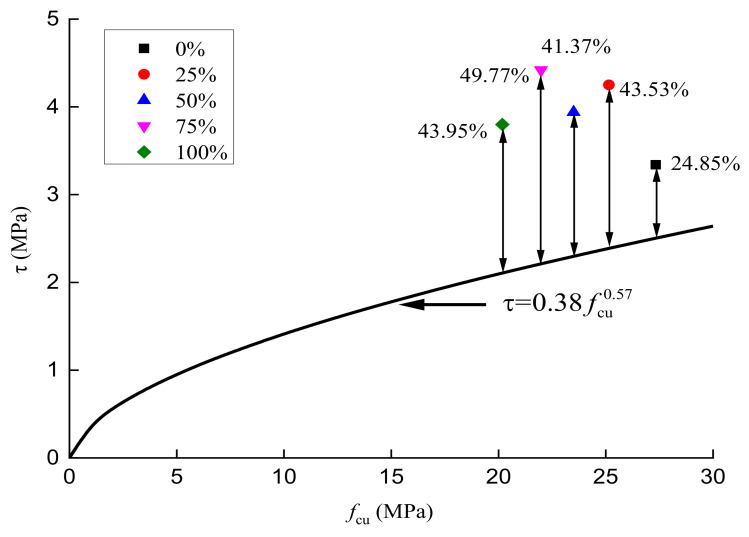
Correlation between the peak compressive stress and peak shear stress of concrete.

Based on the peak compressive stress, peak tensile stress, and peak shear stress of RAC with different substitution ratios, as well as Equations (1) and (2), the relationships among the compressive, tensile, and shear stresses of RAC were obtained, as shown in Figure 7 and Figure 8.

It can be observed from Figure 7 that the relationship between the peak compressive stress and the peak tensile stress of RAC, with five different aggregate substitution ratios, differs from that proposed in reference [41]. When the substitution ratios are 0%, 25%, 50%, 75%, and 100%, the differences between the corresponding measured and calculated values of peak tensile stress of RAC are 31.21%, 26.63%, 24.54%, 22.93%, and 37.12%, respectively. The possible reasons for this relate to the fact that the relationship between the peak compressive stress and peak tensile stress of concrete in reference [39] is proposed based on the experimental data of ordinary concrete. At the same time, the peak tensile stress is obtained by means of direct tension loading, splitting tensile loading, and flexural loading. Furthermore, the maximum dispersions for the peak compressive stress and peak tensile stress of concrete measured are about 15% due to the randomness and discreteness of concrete. In view of the coupling effect of the above factors, the correlation between the peak compressive stress and peak tensile stress of RAC with different substitution ratios obtained in this paper are slightly different from that obtained by the empirical calculation formula in reference [41].

Figure 8 shows that the relationship between the peak compressive stress and the peak shear stress of concrete. According to the test results of this paper and the empirical formula from reference [41], when the substitution ratios of recycled aggregate are 0%, 25%, 50%, 75%, and 100%, the differences between the measured and calculated values of the peak shear stress of RAC are 24.85%, 43.53%, 41.37%, 49.77%, and 43.95%, respectively. It is obvious that the measured values of the peak shear stress of concrete with recycled aggregate or of ordinary concrete significantly differ from the calculated ones. This is likely because the shear mechanical performance test is different from the standard tests of compression or tension loading approaches. The rectangular beam direct shear test, notched beam four-point stress test, and Z-shaped single shear surface test are the generally adopted shear loading test methods. However, the shear test results of specimens corresponding to different test methods are significantly different. For example, when different axial pressures are applied to shear specimens, the maximum difference of the peak shear stress of concrete can reach 2–3 times [34]. In general, the direct shear loading method can effectively control the bending effect and rotation effect in the process of shear loading, and the peak shear stress obtained is more accurate than the peak shear stress obtained by indirect measurement techniques such as Z-shaped single shear. However, the physical properties of recycled aggregate used in RAC are different from those of natural aggregate, so there is a great difference in the mechanical interaction between the different types of aggregate under shearing action. Consequently, the difference between the experimental and calculated values of peak shear stress of RAC is larger than that of ordinary concrete.

### 3.2. Compression Size Effect

#### 3.2.1. Failure Mode

**Figure 9:**
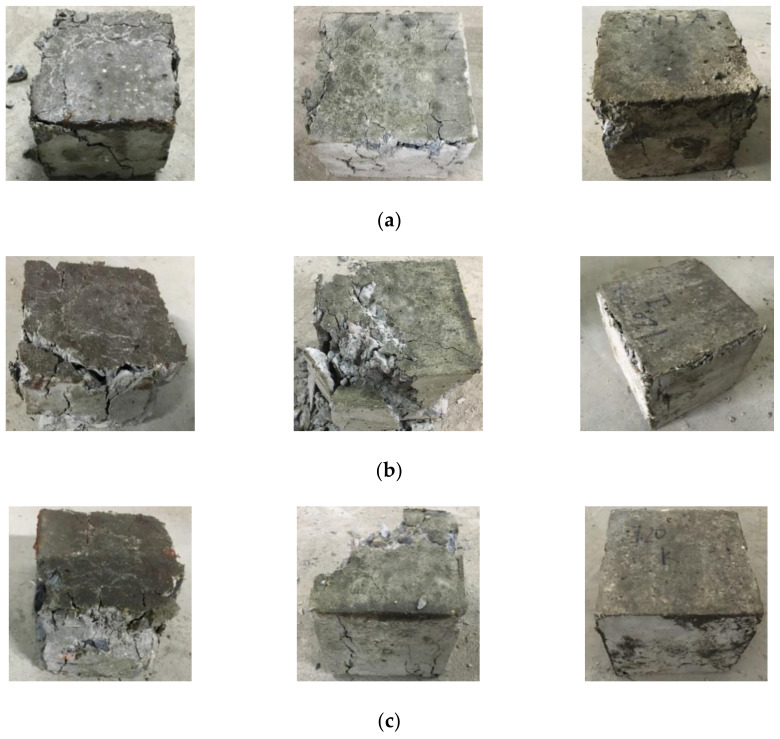
Compression failure modes of the recycled aggregate concrete cubes of various dimensions: (**a**) 0% substitution ratio of recycled aggregate (70 mm, 100 mm, and 150 mm); (**b**) 50% substitution ratio of recycled aggregate (70 mm, 100 mm, and 150 mm); and (**c**) 100% substitution ratio of recycled aggregate (70 mm, 100 mm, and 150 mm).

Based on the experimental scheme for five substitution ratios of recycled aggregate and three kinds of concrete cubes, the failure modes of RAC under different loading modes were obtained. Considering the length of the paper, the failure modes of specimens with different sizes at the substitution ratios of recycled aggregate of 0%, 50%, and 100% were analyzed, as shown in Figure 9.

Analysis of Figure 9 shows that with the same substitution ratio of recycled aggregate, the integrity of RAC after compression failure is largely improved when increasing the side length of the concrete cube. The failure mechanisms for concrete cubes with different side lengths are similar to each other, despite the fact that the cracks in the larger cube specimens are relatively small. When the side lengths of the cube specimens are 70 mm and 100 mm, the integrity of the concrete after compression failure is gradually reduced with the increasing substitution ratio of recycled aggregate. In contrast, when the side length of the cube is 150 mm, the compression failure modes of concrete with different substitution ratios are roughly the same.

#### 3.2.2. Characteristic Value of Peak Compressive Stress

Based on the loading scheme for the compression size effect of RAC with different substitution ratios, the peak compressive stresses of RAC under five substitution ratios of recycled aggregate (0%, 25%, 50%, 75%, and 100%) and three side lengths (70 mm/100 mm/150 mm) cubes were obtained, as shown in Table 4.

It can be understood from Table 4 that the peak compressive stresses of RAC cubes with three side lengths all decrease gradually with a rising substitution ratio of recycled aggregate. When the cubes’ side lengths are 70 mm, 100 mm, and 150 mm, the maximum reduction in the peak compressive stress of concrete is 29.78%, 26.16%, and 20.74%., respectively, by the substitution ratio of RAC. As the side length of the concrete cubes rises, the increase in the peak compressive stress of concrete by the substitution ratio declines progressively. When the substitution ratio of the recycled aggregate is constant, the peak compressive stress of RAC reduces gradually with an increasing side length of the concrete cube. Under the influence of an increasing side length of the concrete cube, the peak compressive stress of RAC decreases by 18.97%, 12.42%, 14.59%, 11.21%, and 8.53%, respectively, at the substitution ratios of 0%, 25%, 50%, 75%, and 100%. Overall, the impact of the size effect on the peak compressive stress of RAC gradually decreases along with an increase in the substitution ratio. The mechanical properties of RAC with different substitution ratios are impacted by the size effect.

In order to further quantitatively analyze the size effect law of RAC with different substitution ratios, this paper refers to the quantitative expression of the size effect presented in the relevant literature [24], and introduces the definition of the size effect degree γ; that is, the larger the size effect degree γ, the more obvious the impact of the size effect on concrete. In this study, the three size lengths for the test on the size effect of RAC cubes were 70 mm/100 mm/150 mm. The test result of peak compressive stress for the RAC cube of 70 mm × 70 mm × 70 mm is taken as the benchmark analysis value to investigate the effect of different side lengths on the peak compressive stress of concrete. The size effect degree γ is defined by Equations (3) and (4):

(3) $$\gamma_{100}=\frac{f_{\mathrm{cu},100}-f_{\mathrm{cu},70}}{f_{\mathrm{cu},70}}\times 100\%$$

(4) $$\gamma_{150}=\frac{f_{\mathrm{cu},150}-f_{\mathrm{cu},70}}{f_{\mathrm{cu},70}}\times 100\%$$

where *f*_cu,70_, *f*_cu,100_, and *f*_cu,150_ are the peak compressive stresses of concrete cube specimens whose sizes are 70 mm/100 mm/150 mm, respectively; the unit is MPa, and γ_100_ and γ_150_ are the size effects of 100 mm/150 mm concrete cubes, respectively.

According to Equations (3) and (4), as well as the test values of the peak compressive stress of RAC listed in Table 4, the size effect degrees γ for concrete cubes of different side lengths were obtained, as shown in Table 5.

It can be observed from Table 5 that when the substitution ratios of recycled aggregate are 0%, 25%, 50%, 75%, and 100%, the size effect degree of a 100 mm × 100 mm × 100 mm concrete cube is 13.03%, 7.87%, 9.06%, 6.32%, and 7.48%, respectively, and that of a 150 mm × 150 mm × 150 mm concrete cube is 18.97%, 2.42%, 14.59%, 11.21%, and 8.53%, respectively. The general trend shows that with an increase in the substitution ratio of recycled aggregate, the peak compressive stress of RAC declines gradually under the impact of the size effect, despite a few fluctuations. This is mainly caused by the randomness and discreteness of concrete materials, which exert little influence on our research results on the size effect.

With a decrease in the substitution ratio of recycled aggregate, the size effect of the peak compressive stress of concrete is gradually enhanced. The principal reason for this is that the initial injuries inside the RAC progressively increase with the rising substitution ratio. Compared with ordinary concrete, RAC exhibits a strong brittleness, which leads to an increase in the substitution ratio of recycled aggregate, thereby further reducing the impact of the size effect on the peak compressive stress of RAC. Our research conclusion is in line with the findings regarding the change law of the size effect on the peak compressive stress of ordinary concrete proposed in references [42,43]; that is, with an increase in the measured peak compressive stress of concrete, the size effect on the peak compressive stress improves gradually.

#### 3.2.3. Law of the Size Effect

According to the basic theory of the size effect established based on the energy release criterion, the size effect of quasi-brittle materials is mainly caused by the strain energy consumed by the generation and expansion of macroscopic cracks inside the materials under external loads. Based on the two conditions of energy balance and deformation coordination, Bazant [44] proposed the expression of the peak compressive stress of concrete, as shown in Equation (5) below:

(5) $$\sigma_{N}=\sigma_{\infty }\times (1+\frac{D_{b}}{D})$$

where σ_N_ is the peak compressive stress of concrete, unit: MPa; σ_∞_ is the nominal peak compressive stress of concrete, unit: MPa; D is the side length of the concrete specimen, unit: mm; and D_b_ is the corresponding characteristic dimension of the structure, unit: mm. In Equation (5), σ_∞_ and D_b_ are the undetermined parameters of the equation.

**Figure 10:**
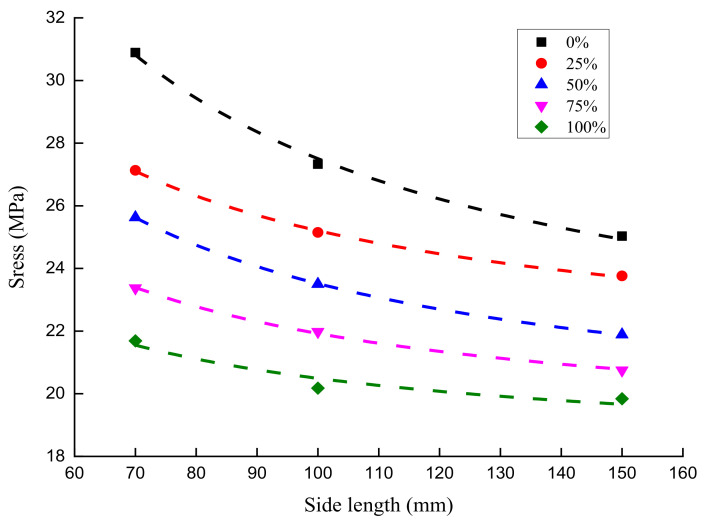
Mathematical regression curve of the size effect law of recycled aggregate concrete with different substitution ratios.

With the peak compressive stresses of different concrete cubes, mathematical regression analysis was carried out through Equation (5). The results show that when the substitution ratios of recycled aggregate are 0%, 25%, 50%, 75%, and 100%, the corresponding values of parameter σ_∞_ are 19.78, 20.77, 18.61, 18.49, and 18.01, respectively, and the values of D_b_ are 39.02, 21.34, 26.04, 18.54, and 13.76, respectively. The size effect law of RAC with different substitution ratios of recycled aggregate are shown in Equations (6)–(10) and Figure 10:

(6) $$0\%:\;\sigma_{N}=19.78\times (1+\frac{39.02}{D})$$

(7) $$25\%:\;\sigma_{N}=20.77\times (1+\frac{21.34}{D})$$

(8) $$50\%:\;\sigma_{N}=18.61\times (1+\frac{26.40}{D})$$

(9) $$75\%:\;\sigma_{N}=18.49\times (1+\frac{18.54}{D})$$

(10) $$100\%:\;\sigma_{N}=18.01\times (1+\frac{13.76}{D})$$

**Figure 11:**
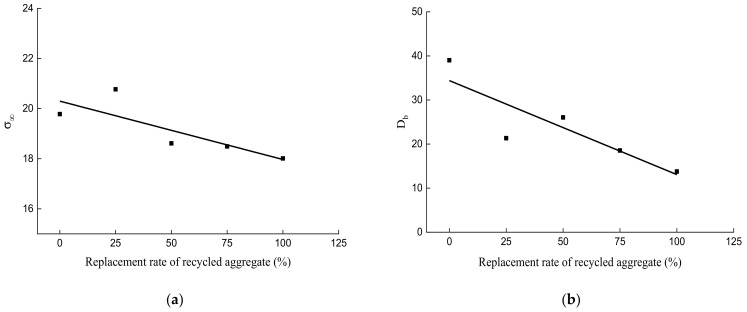
The correlation between the substitution ratio of recycled aggregate and the different parameters: (**a**) parameter σ_∞_; and (**b**) parameter D_b_.

Based on the size effect law, this paper proposes a formula for calculating the size effect on the compressive peak stress of RAC with different substitution ratios, which has good applicability and can better describe the size effect law of the compressive peak stress of RAC with different substitution ratios. To investigate the impact of the coupling effect of the substitution ratio of recycled aggregate and the side length of the specimen on the peak compressive stress of RAC, the influence law of the substitution ratio for parameters σ_∞_ and D_b_ was studied first. Equations (11) and (12) and the expressions shown in Figure 11 are proposed based on the general descending trend of the influence law:

(11) $$\sigma_{\infty }=20.296-0.02328\times \xi$$

(12) $$D_{b}=34.404-0.21328\times \xi$$

It can be observed from Equations (11) and (12) and Figure 11 that our influence equations of σ_∞_ and D_b_ by the substitution ratio of recycled aggregate can explain the relationship between the parameters well. The following expression is obtained by substituting Equations (11) and (12) into Equation (5):

(13) $$\sigma_{N}=\left(20.296-0.02328\times \xi \right)\times \left[1+\frac{\left(34.404-0.21328\times \xi \right)}{D}\right]$$

where ξ denotes the substitution ratio of recycled aggregate, unit: %; and D is the side length of the concrete cube, unit: mm.

Equation (13) is the calculation equation for the peak compressive stress of RAC under the coupling effect of the substitution ratio of recycled aggregate and the side length of the concrete cube. According to the application range of the size effect of practical engineering concrete, when the impact of the size effect on the peak stress of concrete varies within 3%, the critical point for the stress state is called critical stress. The side length of the concrete cube corresponding to the critical stress is the critical dimension. Through Equation (13), the critical dimensions and critical stresses of RAC with different substitution ratios were obtained. The results show that when the substitution ratios are 0%, 25%, 50%, 75%, and 100%, the corresponding critical dimensions of concrete are 1026 mm, 882 mm, 732 mm, 578 mm, and 418 mm, and the critical stresses are 20.98 MPa, 20.36 MPa, 19.75 MPa, 19.14 MPa, and 18.53 MPa, respectively. Therefore, the conclusion that the critical dimension and critical stress of RAC both reduce gradually with the rising substitution ratio of recycled aggregate can be drawn.

**Figure 12:**
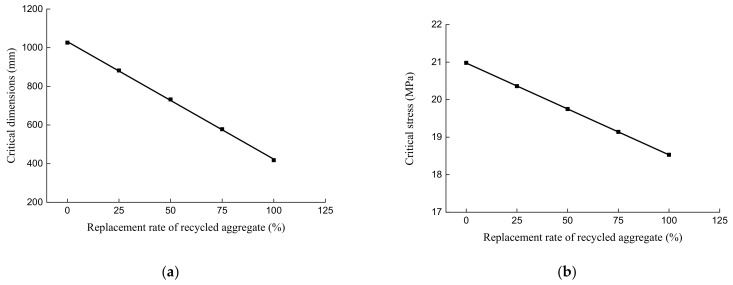
Correlation between the critical dimension and critical stress of recycled aggregate concrete: (**a**) critical dimension; and (**b**) critical stress.

By qualitatively analyzing the critical dimension D_L_ and critical stress σ_L_ impacted by the substitution ratio of the recycled aggregate ξ, Equationss (14) and (15) and the quantitative expressions shown in Figure 12 are proposed:

(14) $$D_{L}=1031.2-6.08\times \xi$$

(15) $$\sigma_{L}=20.976-0.02448\times \xi$$

It can be known from the above formulae and figure that the critical dimension and critical stress of RAC decrease almost linearly with an increase in the substitution ratio of recycled aggregate. The proposed formulae have a good applicability to the quantitative description of the critical dimension and critical stress of RAC affected by the substitution ratio of recycled aggregate.

## 4. Conclusions

In this study, the basic mechanical properties of RAC with different substitution ratios under compression, tension, and shear actions, as well its compression size effects, were experimentally studied. By comparing and analyzing the failure modes and mechanical characteristic parameters of RAC under different loading conditions, the following conclusions can be drawn:

1. With an increase in the substitution ratio of CRCA, the cracks formed on the non-loaded surface of concrete under compression failure mode gradually decline, whereas the tensile and shear failure sections of concrete are relatively unsmooth, accompanied by many concrete fragments, and their failure mechanisms are not affected by the substitution ratio of CRCA. As the specimen’s size increases, the integrity of concrete after the compression failure is progressively enhanced. The substitution ratio of CRCA has the smallest impact on the concrete’s compression failure when the side length of the concrete cube is 150 mm;

2. The load–deformation curves of RAC with different substitution ratios of CRCA were basically the same in compression, splitting tensile, and shear. Additionally, the splitting and shear loading modes showed relatively obvious brittleness characteristics. With the increase in substitution ratio, the peak compressive stress and peak tensile stress of RAC decrease gradually, and the splitting limit displacement decrease, and the splitting tensile modulus increase slightly. Based on the relationship equation of the peak compressive stress, peak tensile stress, and peak shear stress of ordinary concrete, the compressive parameters of RAC with different substitution ratios were compared and the compressive performance of RAC was analyzed from a mechanism perspective;

3. The size effect of RAC under compression was analyzed based on size effect degree. With the same substitution ratio of CRCA, the peak compressive stress of concrete decreases gradually with an increase in the side length of the concrete specimen. As the substitution ratio of CRCA increases, the impact of the size effect on the peak compressive stress of concrete declines gradually. The influence of size effect on the compressive strength of RAC is higher than that of ordinary concrete. Specifically, when the substitution ratios were 0% and 100%, the peak compressive stresses of RAC reduced by the influence of the size effect were up to 18.97% and 8.53%, respectively;

4. Based on the law of the size effect, this paper proposes a calculation equation for the coupling effect of the substitution ratio of RAC and the size effect on the peak compressive stress of RAC. The proposed equation has good applicability. The analysis of the equation revealed that the critical dimension and critical stress of RAC both decline gradually with a rising substitution ratio of CRCA. The research results are of great significance to the application of RAC in engineering projects.

## Figures and Tables

**Table 1:** Physical properties of natural coarse aggregates (NCAs) and coarse recycled concrete aggregates (CRCAs).

Aggregate Type	Bulk Density (kg/m^3^)	Apparent Density (kg/m^3^)	Water Absorption (%)	Mortar Content (%)	Crush Index (%)
NCA	1520	2830	0.43	0.00	4.32
CRCA	1230	2480	3.21	16.28	21.26

**Table 2:** Mix proportions of recycled aggregate concrete (RAC) with different coarse aggregate substitution ratios (unit: kg/m^3^).

Substitution Ratio of CRCA	Water	Cement	Sand	Coarse Aggregate	
				NCA	CRCA
0%	175	461	512	1252	0
25%	175	461	512	939	313
50%	175	461	512	626	626
75%	175	461	512	313	939
100%	175	461	512	0	1252

**Table 3:** Peak compressive, tensile, and shear stresses of recycled aggregate concrete with different substitution ratios (unit: MPa).

Substitution Ratio of Recycled Aggregate	Compressive Stress		Tensile Stress		Shear Stress	
	Peak	SD *	Peak	SD *	Peak	SD *
0%	27.33	2.32	1.73	0.13	3.34	0.25
25%	25.15	2.16	1.69	0.15	4.25	0.31
50%	23.50	1.95	1.63	0.16	3.94	0.29
75%	21.98	1.73	1.57	0.14	4.42	0.41
100%	20.18	1.84	1.32	0.13	3.80	0.33

* Compressive, tensile, and shear stresses standard deviation.

**Table 4:** Experimental values of peak compressive stress of recycled aggregate concrete with different aggregate substitution ratios (unit: MPa).

Cube’s Side Length	Aggregate Substitution Ratios									
	0%		25%		50%		75%		100%	
	Peak	SD *	Peak	SD *	Peak	SD *	Peak	SD *	Peak	SD *
70 mm	30.89	2.83	27.13	2.25	25.63	2.31	23.37	1.85	21.69	1.26
100 mm	27.33	2.32	25.15	2.16	23.50	1.95	21.98	1.73	20.18	1.84
150 mm	25.03	2.12	23.76	1.83	21.89	2.07	20.75	1.72	19.84	1.53

* Compressive stresses standard deviation.

**Table 5:** Strength size effect of recycled aggregate concrete.

Substitution Ratio	0%		25%		50%		75%		100%	
	Peak	SD.	Peak	SD.	Peak	SD.	Peak	SD.	Peak	SD.
γ100 (%)	13.03	1.11	7.87	0.68	9.06	0.75	6.32	0.50	7.48	0.68
γ150 (%)	18.97	1.61	12.42	0.96	14.59	1.38	11.21	0.93	8.53	0.66

## Data Availability

Data available in a publicly accessible repository.
